# Mental health capacity building in refugee primary health care settings in Sub-Saharan Africa: impact, challenges and gaps

**DOI:** 10.1017/gmh.2018.19

**Published:** 2018-08-28

**Authors:** C. Echeverri, J. Le Roy, B. Worku, P. Ventevogel

**Affiliations:** 1 Global Mental Health Consultant, New York, USA; 2 Global Mental Health Consultant, Leuven, Belgium; 3 Global Mental Health Consultant, Department of Psychiatry, School of Medicine, College of Health Sciences, Addis Ababa University, Addis Ababa, Ethiopia; 4 United Nations High Commissioner for Refugees, Public Health Section, Geneva, Switzerland

**Keywords:** Capacity building, impact, interventions, mental health, primary health care, refugee settings

## Abstract

**Background.:**

In 2015, the United Nations High Commissioner for Refugees started a process of mental health capacity building in refugee primary health care settings in seven countries in Sub-Saharan Africa, ultimately aiming to decrease the treatment gap of mental, neurological and substance use (MNS) conditions in these operations. In 2015 and 2016, a specialized non-governmental organization, the War Trauma Foundation, trained 619 staff with the mental health gap action programme (mhGAP) Humanitarian Intervention Guide (HIG), a tool designed to guide clinical decision making in humanitarian settings.

**Methods.:**

This paper describes the results of a process evaluation of a real-life implementation project by an external consultant, one and a half years after starting the programme.

**Results.:**

The mhGAP-HIG capacity building efforts had various effects contributing to the integration of mental health in refugee primary health care. Facility-and community-based staff reported strengthened capacities to deliver mental health and psychosocial support interventions as well as changes in their attitude towards people suffering from MNS conditions. Service delivery and collaboration amongst different intervention levels improved. The scarcity of specialized staff in these settings was a major barrier, hindering the setting-up of supervision mechanisms.

**Conclusion.:**

Mental health training of non-specialized staff in complex humanitarian settings is feasible and can lead to increased competency of providers. However, capacity building is a ‘process’ and not an ‘event’ and mhGAP trainings are only one element in a spectrum of activities aimed at integrating mental health into general health care. Regular supervision and continuing on-the-job training are in fact critical to ensure sustainability.

## Background

Most refugees reside in low and middle income countries where mental services often fall short due to lack of specialized human resources and insufficient funding to set up specialized mental health services (Saxena *et al*., 2007; Kakuma *et al*., 2011). Refugees are at increased risk for developing mental health problems due to a range of risk factors including experiences of violence and upheaval in their home countries, hardships during the flight and ongoing adversities and disrupted social support mechanisms in refugee settlements (United Nations High Commissioner for Refugees, 2015; Silove *et al*., 2017). In 2013 the UNHCR, the UN agency tasked with the protection of and assistance to refugees, issued guidance to assist refugee operations to strengthen services for mental health and psychosocial support (MHPSS) (United Nations High Commissioner for Refugees, 2013). This document is inspired by interagency policy documents such as the ‘Guidelines for Mental Health and Psychosocial Support in Emergency Settings’ by the Inter-Agency Standing Committee (Inter-Agency Standing Committee, 2007). Key elements of these guidance documents are the integration of mental health care within general health settings, as is being promoted by the mental health gap action programme (mhGAP) of the World Health Organization (WHO) (World Health Organization, 2008). An analysis of records from 90 refugee camps showed that mental health care is not sufficiently provided in many refugee settings with significant disparities between camps (Kane *et al*., 2014).

To address these gaps in service provision, the WHO and UNHCR developed the mhGAP Humanitarian Intervention Guide (World Health Organization & United Nations High Commissioner for Refugees, 2015). This practical tool is meant to enable health-care providers in assessing and offering first-line management of MNS conditions in humanitarian emergencies, including refugee camps. The new guide is adapted from the mhGAP Intervention Guide (World Health Organization, 2010), a widely-used evidence-based manual for the management of these conditions (Keynejad *et al*., 2018). Main differences with the regular Intervention Guide are that the humanitarian guide is more concise so it can be used in brief trainings and that it pays particular attention to issues that are relevant to humanitarian settings such as acute stress, grief and posttraumatic stress disorder (Ventevogel *et al*., 2015).

In 2015, immediately after the release of the mhGAP Humanitarian Intervention Guide (mhGAP-HIG), UNHCR engaged a specialized non-governmental organization, the War Trauma Foundation (WTF), to conduct capacity building in mental health in refugee primary health care settings in seven countries in Sub-Saharan Africa where the needs were highest. The primary objective of these activities was to strengthen the capacities of staff from UNHCR and partner organizations to adequately identify refugees with mental, neurological and substance use (MNS) conditions and subsequently provide appropriate and accessible care. Ultimately, it was expected that the capacity building process would contribute in decreasing the burden of mental health problems in poor resource refugee settings.

Within this framework, trainings of 4–5 days based on the mhGAP-HIG were held in 2015 in Cameroon (97 participants), Chad (105 participants) and Ethiopia (66 participants). In 2016, additional courses were organized in Democratic Republic of Congo (51 participants), Kenya (76 participants), Uganda (100 participants) and Tanzania (100 participants). In addition, Training of Trainers and Supervisors (TOTS) sessions took place in Cameroon (24 participants), Chad (25 participants), Ethiopia (16 participants) and Uganda (11 participants) (Table 1).

**Table 1.** Numbers of participants at the mhGAP basic training and TOTS sessions

	Cameroon	Chad	DRC	Ethiopia	Kenya	Uganda	Tanzania	TOTAL
**Basic Training**								
Clinicians	62	50	26	36	30	11	46	
Community workers	35	55	25	30	46	89	54	
**SUBTOTAL**	**97**	**105**	**51**	**66**	**76**	**100**	**100**	**595**
**TOTS**								
Clinicians	24	25		16		11		**76**
**TOTAL**	**97**	**105**	**51**	**82**	**76**	**111**	**100**	619*

* In most of the countries the majority of participants attending the TOTS sessions took part in the basic training sessions

Trainees were divided into two groups: ‘clinicians’ (physicians, clinical officers, general nurses, psychiatric nurses and psychologists where available) and ‘community-based workers’ (mostly refugee ‘incentive’ workers with heterogeneous education levels, together with other humanitarian staff such as community-based psychosocial workers who were not based in health facilities). Trainings for both clinicians and community workers were based on the mhGAP-HIG. Since no community workers’ version of the mhGAP-IG or mhGAP-HIG is yet available, the facilitators had to adapt the contents of the mhGAP-HIG to the community workers’ role, in particular in terms of the skills required for psychosocial interventions.

All clinicians spoke French or English, which allowed using the respective versions of the mhGAP-HIG. Some of the community workers, refugees themselves, had limited knowledge of these two languages; the training courses were thus translated to their native language by other community workers.

In addition to the mhGAP-HIG modules, the courses paid specific attention to topics such as collaborative work based on a multilevel intervention approach and context-specific referral systems.

One and a half year after the first training started, an external consultant (CE) was contracted by UNHCR to assess the effects of the capacity building process, in particular in terms of knowledge retention, attitude change and changes in service delivery.

This paper describes and analyses how the capacity building programme was perceived among trainees and other local stakeholders and evaluates its effects, in an attempt to formulate lessons learnt that may benefit humanitarian operations elsewhere. While various publications have described mental health capacity building in humanitarian settings using the regular mhGAP-IG and other tools (Budosan, 2011; Humayun *et al*., 2017), this is, to our knowledge, the first documentation of such a process using the mhGAP-HIG.

## Methods

A mostly qualitative process evaluation of a real-life implementation project was carried out, using tools such as a desk review of documents (including the facilitators’ reports from the different trainings), telephone interviews with the UNHCR public health officers from the different countries, analysis of the UNHCR's Health Information System (HIS) data and questionnaires filled by some of the trained clinicians in the seven countries, as well as the community workers from Cameroon and Tanzania. These self-reported questionnaires aimed to assess aspects of practice that had changed with the training as perceived by the participants, such as modified attitude towards people with MNS conditions, numbers of patients identified and treated, assessment, diagnostic and management skills (regarding pharmacological and psychosocial interventions for clinicians and only psychosocial interventions for community workers). Likewise, items evaluating the trainee's perception on improved coordination among providers and stakeholders and increased mental health awareness in the refugee setting were included in the questionnaires. Finally, the trainees were asked to provide recommendations to improve the capacity building process as well as to enhance care for people suffering from MNS conditions in their work environment.

As part of the evaluation, the consultant carried out two field visits to the UNHCR's operations in Tanzania and Cameroon. During these field visits additional, again mainly qualitative methods were used:

- Focus group discussions with clinicians (8) and community workers (5) on the perceived effects, challenges and gaps of the capacity building process. The global knowledge retention of the main contents of the training (including the principles of psychosocial intervention, identification of main MNS conditions and related psychoeducation strategies) was also assessed during discussions held with the community workers in Tanzania. Practical questions on signs and symptoms suggestive of prevalent MNS conditions and appropriate psychosocial interventions were asked to this group of trainees.
- Observation of clinical encounters by the clinicians (4) and home visits of refugee families by the community workers (4).
- Discussions with health and mental health managers and coordinators of the different organizations involved.
- Focus group discussions with refugees (3) on levels of satisfaction regarding the available MHPSS services.
- Visits to inpatient departments of the health facilities, interviews with patients and relatives and review of clinical files.

## Results

Various effects were found at different levels of the MHPSS programs of the countries that have been assessed:

## Effects on MHPSS capacities of the service providers

Most of the facility-based staff reported improved clinical skills as shown by the questionnaires they were asked to fill; an average of 81% of the clinicians completely agree that their assessment, diagnostic and management skills have improved (Table 2). The latter was also corroborated in interviews with non-trained colleagues and managers in some countries (Democratic Republic of Congo- DRC, Tanzania). As an example, one of the health centres in Cameroon reports a decreased consumption of analgesics since the training, which has been linked to the improved diagnosis of patients presenting with medically unexplained somatic complaints, who are now receiving psychological rather than pharmacological treatment.

**Table 2.** Perception of enhanced clinical skills, improved coordination among stakeholders and increased visibility of mental health in clinicians and community workers

Clinicians all countries	totally disagree	disagree	neutral	partially agree	completely agree
Perception of enhanced clinical skills*					
Assessment skills	0%	0%	2%	13%	85%
Diagnostic skills	0%	0%	4%	11%	85%
Pharmacological intervention skills	0%	4%	2%	13%	81%
Psychosocial intervention	0%	2%	7%	19%	72%
Perception of better coordination**	0%	11%	4%	13%	72%
Perception of increased mental health awareness***	0%	0%	2%	11%	87%
Community workers Cameroon and Tanzania	totally disagree	disagree	neutral	partially agree	completely agree
Perception of better coordination	0%	0%	3%	10%	87%
Perception of increased mental health awareness	0%	0%	0%	0%	100%

* As assessed through the question: “The training has increased your assessment, diagnostic and pharmacological/psychosocial management skills”

** “The training has improved coordination of stakeholders and providers”

***”The training has increased mental health awareness/brought visibility to mental health in the refugee camp/facility/community you work in”

Similarly, the quality of psychotropic prescriptions by the physicians has improved in some countries (DRC), where UNHCR's public health officer reports that prescriptions are more rational and physicians are for instance using more psychosocial interventions for stress-related conditions.

Overall, clinicians identify and treat more persons suffering from MNS conditions, as reported in the questionnaires (Table 3). 81% of the professionals from this group consider they identify and treat more cases than before the training. In some settings, this reflects in decreased referrals to specialists (Kenya), whereas in countries like Ethiopia, the prescribing skills of general health staff are still insufficient and they continue referring most of the cases for specialized care. In addition, the availability of psychiatric nurses and health officers with a master's degree in mental health in many health facilities in Ethiopia makes referrals an easier option.

**Table 3.** Perception among clinicians and community workers of increased identification and management of MNS conditions and change of attitude

	Cameroon	Chad	DRC	Ethiopia	Kenya	Tanzania	All countries
	Clinicians n = 16	Clinicians n = 11	Clinicians n = 7	Clinicians n = 3	Clinicians n = 3	Clinicians n = 14	Clinicians n = 54
Increased identification of MNS conditions*							
More than before	88%	91%	57%	33%	67%	93%	81%
Same as before	12%	9%	43%	67%		7%	17%
Less than before					33%		2%
Increased treatment of MNS conditions**							
More than before	94%	91%	57%	33%	67%	86%	81%
Same as before	6%	9%	14%	67%		14%	13%
Less than before			29%		33%		6%
Attitude change towards people with MNS conditions***	100%	100%	100%	100%	100%	100%	100%
Increased identification of MNS conditions*	Community Workers n = 7					Community Workers n = 23	Community Workers n = 30
More than before	100%					96%	97%
Same as before						4%	3%
Less than before							
Attitude change towards people with MNS conditions***	100%					100%	100%

* As assessed through the question: “The number of patients with MNS conditions you identify is less, same, more than before the training?”

* “The number of patients with MNS conditions you treat/manage is less, same, more than before the training?”

*** “Do you think your attitude towards people with MNS conditions has changed in terms of empathy, openness, tolerance, communication style?”

The findings among the community workers’ group are somewhat less consistent, differing across the operations as well as inside the teams. Most of them perceive their capacities as strengthened in terms of case identification, referrals and psychoeducation: 97% of the community based-staff who filled the questionnaires in Cameroon and Tanzania report identifying more people suffering from MNS conditions (Table 3). However, some community workers state they manage fewer cases than before the training and that they refer them to the health centres (Tanzania), whereas in Cameroon most of them report providing more psychosocial interventions than before the course. The latter finding in Tanzania could be related to the improved identification of disorders needing medical or psychological interventions and hence increased referrals, but could also be due to insufficient psychosocial intervention skills leading to a referral of cases that could be eventually managed at the community worker's level. It is important to note that the community workers who were part of the training had variable educational status and experience at baseline, which could also affect their performance after the training.

A good basic knowledge of the psychosocial intervention principles was observed among community workers during the assessment in Tanzania.

Analysis of the mhGAP pre-and post-tests generally showed score increases, particularly among clinicians; these changes tended to be more significant among trainees with weak MHPSS capacities before the course, such as nurses. These WHO questionnaires aim testing the capacities of the clinicians and community workers before and after the training. The tests assess three types of skills in MHPSS providers in humanitarian settings: knowledge on MNS conditions, participants’ perception on their assessment and management skills (technical skills) and capacities to make a multi-level and integrative care management plan for a concrete case (case management). The results of the pre- and post-tests were discussed and reported back to the participants individually and at the group level. This evaluation strategy allowed both participants and trainers to appreciate the progress made and to identify remaining gaps.

**Fig. 1.**
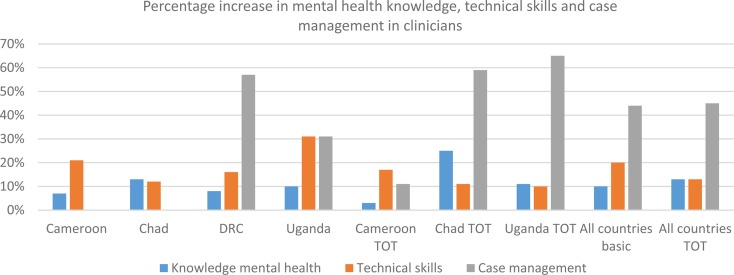
Average percentage increase in mhGAP HIG post-test scores in clinicians attending basic and refresher/TOTS training.

A significant improvement on the three types of skills was found among clinicians and community workers, in both the basic and refresher/TOTS courses (Fig. 1). The available data from Cameroon, Chad, DRC and Uganda showed an average increase of around 10% in mental health knowledge, 15% average increase in technical skills in clinicians/20% in community workers and 44/53% improvement in case management skills. It is noteworthy that the facilitators considered the case management skills as most relevant when assessing progress, as these reflect the competencies in assessing a patient and designing a management plan integrating both health facility-based clinical care and community-based support (pharmacological and psychosocial components of care).

Score improvement differed from country to country as well as among trainees of the same group, which can be explained by the participant's varying levels of competencies, related to their education level and previous experience in MHPSS work. In addition, some language issues were reported in groups where participants did not speak the same language as the trainers, which hindered the learning process. Moreover, some of the trainers highlighted that several pre-and post-test questions were not adapted to the community workers’ profiles and that scores did not reflect changes in practical skills (Tanzania). Questions about the validity and reliability of these tests when measuring knowledge and skills improvement have been raised by the trainers and it is important to keep in mind that improved post-test scores do not necessarily correlate with lasting knowledge retention.

## Effects on service utilization

The analysis of the UNHCR's Health Information System (HIS) data yielded inconclusive results: in some countries, the numbers of monthly consultations for some MNS conditions increased after the training (Tanzania), whereas in others the numbers decreased (Kenya), remained stable or fluctuated over time (DRC).

Increased numbers could be explained by strengthened diagnostic skills of the health staff with improved registration of cases according to the HIS categories. In addition, intensified mental health activities including community sensitization could also increase the numbers of refugees with MNS conditions seeking medical care. On the other hand, decreased numbers of consultations could reflect contextual factors such as a temporary shortage of psychotropic supplies in the health facilities or fluctuating numbers of ‘incentive’ community workers due to the displacement of refugees, all of this leading to decreased numbers of patients receiving care. It is important to note that no firm conclusions can be reached from these differences in service utilization due to the qualitative nature of this assessment.

## Attitude towards people with MNS conditions

All of the trainees (clinicians and community workers) who filled in the questionnaires perceive their attitude has positively changed (100%, Table 3), in particular in terms of increased empathy towards people suffering from MNS conditions. Some professionals report practicing more active listening and feeling overall more open, tolerant and less judgemental when interacting with patients with complex problems (Chad, Kenya). This would be related to a better understanding of the patient's underlying condition due to the training, according to some of the trainees (DRC, Kenya). Some community workers highlight the fact that they are paying more attention to confidentiality (Tanzania). Although the subjective nature of these self-report measures do not allow conclusions to be drawn, it is interesting to note that all of the trainees perceived their attitude toward people with MNS conditions had been modified with the training.

## Increased mental health awareness in refugee settings

Building capacities of providers has promoted greater awareness and visibility of mental health at different levels: 87% of clinicians and 100% of community workers completely agree that there is more awareness on the subject thanks to the training (Table 2). As an example, the latest World Mental Health Day celebration in Kakuma refugee camp in Kenya saw the participation of more partners, which has been related to increased MHPSS awareness thanks to the training.

As a result of the increased awareness, stigmatization of people with MNS conditions would reportedly have decreased in some refugee communities (Cameroon). The latter could be also related to improved MHPSS services: effective treatment of people suffering from mental conditions has shown to be a powerful tool for community sensitization.

## Effects on service delivery

The findings suggest that building capacities of the health staff has overall promoted the integration of mental health into primary health care. In some settings the training contributed to improve inpatient care at the general health facilities: in Tanzania, more acute cases are stabilized at one of the refugee health centres thanks to the enhanced skills of the medical doctors in charge, whereas in Cameroon the process has allowed to open mental health units in district hospitals, run by psychiatric nurses who were part of the capacity building process.

The trainings have also catalysed the implementation of referral and supervision systems in some operations (Uganda, Tanzania), which has played a critical role in improving the quality of MHPSS services. Involving local psychiatrists as facilitators of the training, some of them working at nearby psychiatric hospitals, did not only increase the effectiveness of the teaching sessions but also facilitated the establishment of referral systems (Cameroon, DRC, Uganda, Tanzania). Operational referral systems are in fact a favourable space for providers to continue practicing the skills acquired during the training in a multi-layered MHPSS programme.

The experience of Uganda is noteworthy, as it started with a Training of Trainers and Supervisors (TOTS) session followed by a basic course where the same TOTS participants trained general health staff under the supervision of the facilitators’ team. During the process, the TOTS trainees gained in confidence by applying the recently acquired training skills; moreover, they created links with the general health staff they were training, which most probably enhanced collaborative work and facilitated future supervision activities. This particular design of the training had a positive outcome, promoting the rapid establishment of a supervision system, which is a key element for the process’ sustainability. A similar positive experience has been reported after the 2016 TOTS training in Ethiopia.

During the group discussions with refugees in Cameroon and Tanzania many patients’ relatives reported that the quality of MHPSS services had improved and declared being generally satisfied with care.

## Collaboration between the different intervention levels

There is a general perception of improved collaborative work between the different levels of care of the MHPSS systems. The joint training sessions involving community workers, clinicians and sometimes managers largely contributed to this; the different providers have now a better understanding of their respective roles and interact more efficiently (Tanzania).

In countries such as DRC, the training promoted establishing links with traditional healers in the refugee community, who have started referring cases to the health centres after a sensitization/education meeting was held.

Furthermore, the trainings created good opportunities to develop new tools promoting collaborative work. In Ethiopia, a common case registration system used by community and health workers has been created. In northern Uganda, the different MHPSS partners developed and implemented a weekly joint mental health reporting system.

Likewise, MHPSS action plans including referral pathways developed during the training's joint session are starting to be implemented in some settings (DRC, Kenya, Uganda, Tanzania).

Another significant effect of the capacity building process is the establishment of partnerships with national health authorities. In Cameroon, the collaboration between the organization in charge of providing mental health services and the Ministry of Health, and furthermore, the participation of the Ministry's mental health deputy director in the training sessions, catalysed the implementation of the mhGAP approach not only in the refugee-hosting areas but also at a national level. This led for instance to the development of mental health units in general hospitals in several regions, as mentioned before. The availability of such services allows to stabilize acute patients while limiting referrals to the psychiatric hospital in the capital.

## Coordination amongst stakeholders

Although many trainees perceive that coordination among the different MHPSS actors has overall improved (72% of clinicians and 87% of community workers completely agree on this, Table 2), a considerable number of clinicians disagrees or does not have a positive either negative opinion on this subject. These perception variations most probably reflect the challenges of implementing MHPSS systems in refugee settings and in particular putting in place effective coordination mechanisms. It is nevertheless noteworthy that MHPSS coordination mechanisms have been established in most of the assessed operations after the training. Of note, MHPSS working groups established after the mhGAP courses are meeting regularly in settings such as Tanzania and northern Uganda.

## Challenges and barriers

Several challenges and barriers to the capacity building process have been identified:

## Inadequate selection of trainees

Including in the same group participants with knowledge levels and profiles that differ too much was found to hinder the training process (Chad, Ethiopia, Tanzania). An additional problem in some countries like Ethiopia was the underrepresentation of women among the community workers’ group of trainees, which might subsequently impact service delivery, since many female refugees will feel culturally restrained to seek psychosocial support from a male.

Similarly, not including key staff from the different intervention levels (community, primary health facilities, district hospitals and psychiatric hospitals when possible) was found to negatively impact the establishment of operational referral systems.

## High turnover of staff

The facility- and community- based staff regularly changes in refugee settings and the risk of lack of transmission of the acquired knowledge and experience is real. Knowledge and skills transfer and continued training and refresher courses should be included in the UNHCR's partner organizations’ policies.

## Scarcity of specialized staff

In some operation areas, the absence of psychiatrists or psychiatric nurses acts as a major barrier by limiting the possibilities to set-up a supervision system, which, as mentioned before, is a key factor for the sustainability of the process. In many countries (Cameroon, DRC, Ethiopia), the fact that psychiatrists are only available in large cities is a major constraint because of the long distances and costs implied to implement regular supervision visits. In countries such as Chad, the absence of specialized human resources (only one psychiatrist practicing in N'djamena), further limits continued capacity building. Building mental health capacities in nurses is a useful strategy to meet this challenge, as these professionals are usually available in refugee settings and tend to rotate less often than physicians or clinical officers.

## Insufficient involvement of governmental stakeholders

Governmental health staff from the local and central levels was not sufficiently represented in some settings (Chad). Involving clinicians and managers from the public sector was found to contribute to the sustainability of the process (Cameroon).

## Reflections and conclusions

The mhGAP-HIG capacity building efforts had various effects in the different countries where the trainings were held, contributing overall to the integration of mental health in refugee primary health care.

The findings suggest a strengthening of MHPSS capacities of the providers, as reflected in various settings in general health staff treating more persons with MNS disorders and community workers identifying and referring more cases, as well as providing psychosocial support when needed. Nevertheless, knowledge and skills of clinicians and community workers are still insufficient in some countries and continuing capacity building is required. Providers reported positive changes in attitude, in particular in terms of increased empathy towards people suffering from mental health conditions.

In addition, service delivery has improved, as shown by better quality out- and inpatient care in some of the health facilities, which is a clear step forward in the process of scaling up mental health services. The trainings have also catalysed the implementation of referral and supervision systems in some operations, which has played a critical role in improving the quality of services.

Moreover, collaborative work between the different levels of care of the MHPSS systems has generally improved, thanks to a better understanding between community workers and clinicians, who are starting to interact more efficiently. MHPSS action plans including referral pathways developed during the trainings are starting to be implemented in some countries. Furthermore, the capacity building process created and strengthened partnerships with national health authorities in several countries including Cameroon, where this collaboration catalysed the implementation of the mhGAP approach at a national level.

The coordination amongst the different stakeholders has been similarly enhanced, as shown by the establishment of MHPSS coordination mechanisms in most of the operations. Finally, it was found that the process has promoted greater awareness and visibility of mental health in the refugee settings.

At the same time, several challenges and barriers to the capacity building process have been identified. The main challenge found in this evaluation is the general scarcity of specialized staff in Sub-Saharan countries, which represents a major barrier in setting-up supervision mechanisms. Regular supervision with emphasis on on-the-job training has been identified as a critical element for the sustainable building of capacities. In this sense, the Ugandan design of the courses starting with a TOTS session followed by a basic course conducted by the same TOTS trainees has shown to be a good option to rapidly establish a supervision system, provided that mental health professionals such as psychiatric nurses are available. The experience in Cameroon also shows that trained psychiatric nurses can engage in supervision activities in the refugee health centres, ideally supported by regular visits by a psychiatrist.

In addition to regular visits by a specialist, having the possibility of technical support from the supervisor via phone calls or messaging can be of great help for trained general health staff. Additional support options that should be considered are establishing peer support groups and online linkages involving supervisors and trainees, such as social media groups.

Implementing operational referral systems is another factor that contributes to the process by giving the possibility to providers to continue practicing the skills acquired during the training in a multi-layered collaborative MHPSS intervention.

It is important to mention that developing partnerships with the public sector, both at a local level (including governmental health staff in the training sessions) and at central level (involving mental health authorities when possible) is a key strategy to promote sustainability of the capacity building process.

Finally, the evaluation showed that capacity building is a ‘process’ and not an ‘event’ and that mhGAP training can only be one of the elements of a spectrum of activities aimed at the integration of the mental health component in general health care.

The qualitative nature of this evaluation, based primarily on perceptions of change reported by the different actors as well as of direct observation of service provision, does not allow to draw firm conclusions; further quantitative outcome evaluations are required for a better understanding of the impact of such processes. While some results of the evaluation are inconclusive, this paper demonstrates that important lessons can be drawn from proper documentation and external evaluation of multi-country implementation of capacity building in routine refugee health care.
